# Dietary factors associated with metabolic syndrome and its components in overweight and obese Brazilian schoolchildren: a cross-sectional study

**DOI:** 10.1186/s13098-016-0178-9

**Published:** 2016-08-24

**Authors:** Ana Elisa Madalena Rinaldi, Gleice Fernanda Costa Pinto Gabriel, Fernando Moreto, José Eduardo Corrente, Kátia Cristina Portero McLellan, Roberto Carlos Burini

**Affiliations:** 1 School of Medicine, Federal University of Uberlândia (UFU), Rua Pará, nº 1720, Bloco 2U, Uberlândia, MG 38405-320 Brazil; 2 School of Medicine, Southern Paraná State University (Unioeste), Universitária 2069 St., Cascavel, PR 85819-110 Brazil; 3 Member of the Exercise Metabolism and Nutrition Center (CeMENutri), Distrito de Rubião Júnior s/n, Botucatu, SP 18618-970 Brazil; 4 Department of Biostatistics, Institute of Biosciences, Distrito de Rubião Júnior, s/n, Botucatu, SP 18618-900 Brazil; 5 Texas Institute for Kidney and Endocrine Disorders, Texas Institute for Kidney and Endocrine Disorders, 10 Medical Center Blvd, Ste A Medical Center, Lufkin, 75904 USA; 6 Botucatu School of Medicine, Public Health Department and Exercise Metabolism and Nutrition Center, São Paulo State University, Distrito de Rubião Júnior, s/n, Botucatu, SP 18618-970 Brazil

**Keywords:** Metabolic syndrome, Schoolchildren, Dietary intake, Overweight, Obese

## Abstract

**Background:**

The metabolic syndrome (MS) has been assessed since childhood mainly because of the nutritional and epidemiological transition that has occurred worldwide. Our objectives were to explore the MS and its components according to anthropometric and demographic factors and to assess the relationship among MS components and dietary characteristics in overweight and obese schoolchildren.

**Methods:**

This was a cross-sectional study which included 147 schoolchildren (aged 6–10 years) from three elementary schools, with body mass index (BMI) higher than the 85th percentile. Sexual maturation stages, anthropometric measures (weight, height, skinfold thickness and waist circumference), biochemical data (glucose, HDL-C and triacylglycerol), blood pressure and dietary intake were assessed. The metabolic syndrome was diagnosed if three or more of the following components were presented: waist circumference ≥90th age and sex-specific cut-off, blood pressure ≥90th age, sex and height-specific cut-off, glucose ≥100 mg/dL, HDL-C ≥ 40 mg/dL and triacylglycerols ≥ 110 mg/dL. The dietary intake was assessed by three non-consecutive 24-h recalls. The T test, Kruskal–Wallis and multiple linear regression analysis were applied to assess MS components and dietary intake.

**Results:**

The MS percentage was 10.2 % and it was higher in obese children and ones with high body fat percentage. The waist circumference was the main altered component of MS and 62 % of overweight schoolchildren showed at least one altered component of MS. The components of metabolic syndrome associated with dietary intake were triacylglycerol (positive association with saturated and monounsaturated fat, whole-milk products and processed foods and negative associated with legumes and polyunsaturated fat), glycemia (positive association with processed foods and negative with cereals), HDL-C (positive association with vegetables and greens) and waist circumference was negative associated with protein.

**Conclusions:**

The frequency of MS was higher in obese than overweight schoolchildren and the frequency of at least one MS component was high in more than half of our subjects. The waist circumference was the most frequent among all other components. The triacylglycerol and glycemia were the most frequent MS components associated with dietary intake. Unprocessed food was considered a protective dietary factor for MS metabolic components and processed food with high percentage of sugar and saturated fat was a risk factor for MS metabolic components.

## Background

The definition and diagnosis of metabolic syndrome (MS) in pediatric population is still controversial and not yet consensual [1]. The challenge of establishing a definition for MS through childhood is related to many methodological and physiological limitations, such as factors that influence plasma lipid levels (age, sex, and race) [2], presence of a transient physiologic insulin resistance during puberty [3, 4], lack of a standard central obesity measurement, and lack of normal ranges for insulin concentrations. Moreover, children and adolescents with MS may have different threshold values for laboratory abnormalities when compared to adults [5].

There are different metabolic syndrome criteria cut-off values, but there is an agreement for the use of components, such as one anthropometric parameter (waist circumference or body mass index), one parameter for glucose metabolism (HOMA-R or fasting glycemia) and two parameters of lipids (triacylglycerols and HDL-C) and blood pressure [6]. The most used criteria are those proposed by Cook et al. [7] which is an adaptation of the National Cholesterol Education Program—Adult Treatment Panel (NCEP-ATP III) [7] and by international diabetes federation (IDF) [8]. The main difference between them is that IDF established that MS cannot be diagnosed for children under 10 years of age. However, for children with family history of MS, hypertension and diabetes mellitus it should be assessed. Although being of difficult diagnosis, MS in childhood has been supposed to be a risk factor for adult cardiovascular and metabolic complications [2]. The MS components could be selected in studies during childhood as cross-sectional predictors in order to mark the presence of metabolic impairment [2].

Obesity could be considered a key risk factor for the development of MS, mediated mainly by lipid partitioning (distribution of body fat in organs and compartments). The effect of obesity is determined by the pattern of lipid partitioning affecting the secretion profile of adipocytokines and free fat acids. The combined effect of these factors determines the sensitivity of insulin at the target organs (liver and muscle) and the endothelial function. The visceral fat is more resistant to insulin [1]. Unhealthy diet and physical inactivity are considered risk factors for obesity. Obese Mexican schoolchildren showed lower quality of diet, higher intake of sweetened beverages and refined carbohydrates with added fat and they were less physically active at school than eutropic schoolchildren [9].

Some studies have explored the association of dietary intake and MS or its components in children and adolescents [10–13]. High processed food rich in hydrogenated vegetal fat, sodium, refined grains and sugar have been the main focus of studies and it is associated with the increased obesity and comorbidities rates [14–16], and MS [10]. A high sugar-sweetened beverages intake (500 mL/day) was associated with MS in Taiwan adolescent boys. The sugar-sweetened beverages intake is associated with higher waist circumference, systolic blood pressure and triacylglycerols values [17]. There is a positive association between higher intake of high processed food and MS prevalence in Brazilian adolescents [18]. A study with pre-puberty Korean children showed a positive relationship between higher scores of western dietary pattern and the prevalence of MS components [19]. On the other hand, the intake of fruits, vegetables, and dairy products seems to have an inverse association with the presence of MS [11].

The studies about dietary intake and MS previously described included adolescents or pre-adolescents. Our study can contribute to literature because we studied the MS characteristics and its relationship with dietary factors in the beginning and in the end of childhood. We explored the macronutrients and food groups associated with each MS components as well. This study had two objectives: the first one was to explore the MS and its components according to anthropometric and demographic factors and the second one to assess the relationship among MS components and dietary characteristics in overweight and obese schoolchildren.

## Methods

### Subjects and study design

This was a cross-sectional study including 147 overweight and obese schoolchildren aged 6–10 years, from three primary schools of Botucatu, São Paulo State, Brazil. The study was developed between June-2007 and August-2008. These schools were chosen based on previous approval of their principals and the Municipal Department of Education. Parents or legal guardians signed consent forms for their children’s participation on this study. The inclusion criteria consisted of BMI >85th percentile and the absence of any endocrine, renal, heart, or liver diseases. We opted to exclude children with previous diagnosis of diabetes mellitus (type I or II), because it could have altered lipids, blood pressure and mainly food intake. We included only overweight and obese schoolchildren because the excess of body weight and body fat are the main risk factors for high blood lipids, high blood pressure and MS. A recent recommendation of clinical management is that all obese children and adolescents should be assessed for MS components. We applied BMI as a screening tool to select overweight and obese with possible high health risk factor [2, 20, 21].

**Fig. 1:**
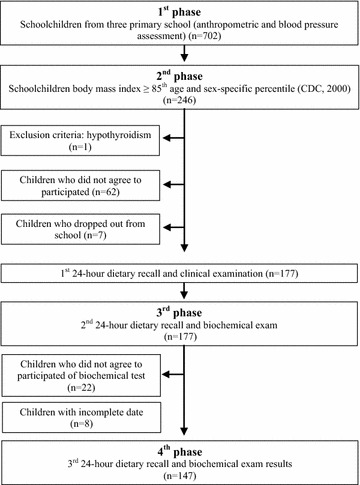
Flowchart of overweight and obese schoolchildren selection (4 phases)

The study was developed in 4 phases: (1st) It was performed the anthropometric and blood pressure assessment screening (n = 702). (2nd) It was performed the selection of eligible schoolchildren (BMI ≥85th age and sex-specific percentile) (n = 246). One child was excluded because of hypothyroidism, 62 schoolchildren did not agree to participate in the next phases of the study, and six schoolchildren dropped out from school. The invitation for all overweight and obese schoolchildren to participate on the three phases of this study was implemented by a phone call for parents or legal guardians. The anthropometric diagnosis was explained (overweight or obese) and they were invited to participate in the following three phases. We opted to call to avoid any type of social exposition of the overweight and obese schoolchildren. After the phone call invitation and the exclusion criteria, it was applied the first 24-h dietary recall and the clinical examination (n = 177). (3rd) A biochemical exams was performed and then the second 24-h dietary recall was applied (n = 155). Twenty two schoolchildren were excluded for not having biochemical exams. (4th) The biochemical exam results were explained to the parents and it was applied the third 24-h dietary recall (n = 147) (Fig. 1). All 24-h dietary recalls, clinical examinations and biochemical exams were developed in each school.

It was relevant to explain that parents/legal guardians from overweight and obese schoolchildren signed two different consent forms: the first one was for allowing schoolchildren to participate on the anthropometric assessment screening and the second one was allowing schoolchildren to participate on the next three phases of the study (only overweight and obese schoolchildren).

### Anthropometric, blood pressure and sexual maturation assessment

Anthropometric status assessment consisted of measuring weight (Filizola^®^), height (Seca^®^), arm and waist circumference and triciptal and subscapular skinfolds (Lange^®^) according to World Health Organization [22]. BMI was calculated by dividing the weight (kg) by the height squared (m^2^) and it was classified according to age and sex-specific percentiles [23]. Body fat percentage was estimated using a sex-specific equation proposed by Slaughter et al. [24], based on the sum of tricipital and subscapular skinfolds. The body fat percentage was classified as moderately high if it was ≥25 % for girls and ≥20 % for boys [25].

Blood pressure was measured using auscultatory method and it was classified according to sex, age and height percentile [26]. Sexual maturation stages were assessed by a pediatrician and classified according to Tanner sexual maturation scale [27]. We classified schoolchildren as pre-pubertal (stage 1) and pubertal (stages 2, 3 and 4). Nobody was classified into stage 5.

### Biochemical tests and the MS

Schoolchildren were submitted to vacuum venous puncture after 12-h nocturnal fasting. Biochemical parameters of glucose, HDL-C and triacylglycerols were quantified by using a semi-automated spectrophotometer (Labquest^®^, Labtest Diagnóstica) and commercial kits (Labtest Diagnóstica) by the enzyme colorimetric method.

The MS was diagnosed according to National Cholesterol Education Program—Adult Treatment Panel III (NCEP-ATP III) criteria with some adaptations proposed by Cook et al. [7] for children and adolescents. Therefore, the MS criteria after all adaptation was: waist circumference ≥90th age and sex-specific cut-off [28], blood pressure ≥90th age, sex and height-specific cut-off [26], glucose ≥ 100 mg/dL [29], HDL-C ≥ 40 mg/dL and triacylglycerols ≥ 110 mg/dL [7].

### Dietary intake assessment

Three 24-h dietary recalls were applied to assess dietary intake (two of them referring to non-consecutive weekdays—and one to the weekend). In addition, all food and beverages consumed on the previous day of the visit were recorded by the interviewer [30]. The 24-h dietary recalls were applied in the presence of any adult responsible for the child’s diet, in order to enhance answer accuracy. Dietary data obtained in homemade measurements were converted into grams and milliliters to estimate the total energy intake, macronutrients and fiber [31–33]. A photographic food atlas of portion size was used to improve the accuracy of food intake [34]. The centesimal composition of food was calculated using NutWin^®^ (2002) software, version 1.5., a Brazilian software that incorporates the United State Department of Agriculture (USDA) nutrient database.

Food and preparation were transformed into servings according to six basic food groups: cereals, legumes, vegetables, fruits, meats and egg (red meat, chicken, fish, and egg), and dairy food (milk, yogurt and cheese). The oil and fat group was expressed as saturated, mono and polyunsaturated, and sugar as a percentage of total energy intake. The following foods were considered in the sugar group: sugar, honey, juice powder, condensed milk, *dulce de leche*, sodas, artificial fruit juice, sweetened beverages, sweetened soy-based fruit beverages, candy, gum, lollypops, chocolate drinks, energy bars. Meal preparations were broken down into ingredients, so that they could be included in their respective food groups.

We assessed the percentage of sugar and processed (industrialized) food from total energy intake and the food was separated into two groups: *processed food* (cake mix, chips, frozen food, nuggets, chocolate drinks, pudding mix, noodles, microwave popcorn, sugar-based breakfast cereal cookies) and *food with high sugar and fat content*—those with a fat percentage higher than 35 % and sugar higher than 10 % of total calories (chocolate, milk-based ice cream, sandwich cookies, wafers).

### Statistical analysis

The normality of data was tested by the Shapiro–Wilk Test. The triciptal skinfold and height were summarized in average and standard deviation. The weight, BMI, waist circumference, subscapular skinfold and body fat percentage were summarized in median and interquartile interval. Sexual maturation and MS components were described in percentages. Student’s Test T and Kruskal–Wallis were used to compare the anthropometric measurements in different MS component numbers. The multiple linear regression model was used to explore the relationship of dietary intake (expressed in macronutrients, food groups and total energy percentage) and the MS components, adjusted for sex, age and school. For such adjustment, the MIXTRAN routine was used. This routine was developed by Tooze et al. [35] and considers a mixed model of two parts: frequency and amount consumed. In this case, only the second part of the model, with the adjustment of the amounts consumed, was used. This routine already considers data normality using the Box-Cox transformation. The level of significance adopted to be considered in the model was 0.05. The level of significance adopted for all statistical analysis was p < 0.05. Statistical analysis was performed using the statistical analysis Software (version 9.1.3, 2006, SAS Institute Inc, Cary, NC).

## Results

One hundred and forty-seven overweight and obese schoolchildren participated in the study, average age of 7.9 ± 1.4 years, 52 % girls and 63 % obese. Most of the schoolchildren (80.3 %) were classified with high body fat percentage and the average values were similar for both sex. Regarding sexual maturation, 66.7 % were classified as pre-pubertal. The average age of pre-pubertal was lower than pubertal, 7.5 ± 1.3 and 8.9 ± 1.0 years old, respectively (data not shown). All anthropometric measures were significantly higher for children with one or more MS components and higher for children with MS. The average age was the same among three categories of MS components (Table 1).

**Table 1:** Age and anthropometric measures of overweight and obese schoolchildren by number of MS components. Botucatu-SP-Brazil, 2007–2008

Anthropometric measures^d^	MS components median (1st; 3rd quartiles)			p trend
	0 (n = 56)	1 and 2 (n = 76)	≥3 (n = 15)	
Age (years)	7.8 ± 1.4^a^	7.9 ± 1.4^a^	8.3 ± 1.7^a^	0.224
Weight (kg)	33.1 (29.7; 38.9)^a^	40.5 (34.6; 49.8)^b^	50.3 (39.0; 61.9)^c^	0.000
Height (cm)	1.31 ± 0.1^a^	1.37 ± 0.11^b^	1.44 ± 0.13^b^	0.000
BMI (kg/m^2^)	19.8 (18.8; 20.9)^a^	22.3 (20.9; 24.2)^b^	25.1 (24.2; 27.4)^c^	0.000
WC (cm)	64.1 (61.3; 69.3)^a^	73.0 (67.5; 78.3)	81.0 (75.0; 87.0)^c^	0.000
Triciptal skinfold (mm)	17.9 ± 3.9^a^	20.8 ± 4.6^b^	23.5 ± 4.0^c^	0.000
Subscapular skinfold (mm)	10.3 (8.0; 14.5)^a^	16.0 (12.0; 20.0)^b^	20.0 (17.0; 23.0)^c^	0.000
Body fat (%)	25.3 (21.9; 29.2)^a^	30.4 (26.1; 33.3)^b^	33.2 (29.4; 38.1)^c^	0.000

*BMI* body mass index, *WC* waist circumference

^a, b, c^Different letters = statistically significant

^d^Height and tricipital skinfold are expressed in average and standard deviation

**Fig. 2:**
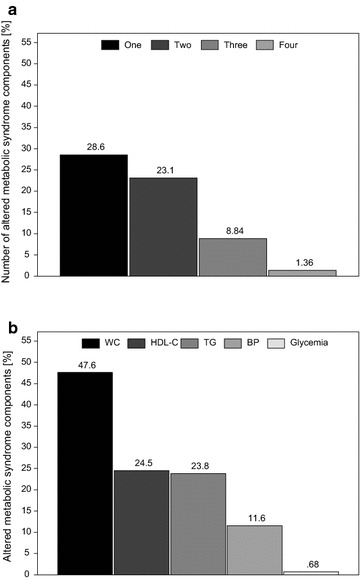
Number of MS altered components (**a**) and type of MS component (**b**) of overweight and obese school children. Botucatu-SP-Brazil, 2007–2008

The percentage of MS in our study was 10.2 %. The percentage of schoolchildren which had one altered MS component was 28.6 % and two was 23.1 % (Fig. 2a). The percentage of MS was similar for sex (6 girls and 9 boys, p value = 0.339) and sexual maturation (10 pre-pubertal and 5 pubertal, p value = 0.604). However, MS was significantly higher for obese (2 overweight and 13 obese schoolchildren, p value = 0.042) and for elevated body fat percentage schoolchildren (0 adequate and 15 elevated body fat percentage, p value = 0.043) (data not shown). The most frequent altered MS component was abnormal waist circumference (47.6 %) followed by lipids (HDL-C and triacylglycerols) (Fig. 2b).

We explored the association among MS components and energy intake, macronutrients, cholesterol and fiber (Table 2) and among MS components and food groups and percentage of energy intake from sugar and processed foods (Table 3). There were not association among MS and dietary characteristics. One possible reason was the low percentage of MS in this sample. The multiple linear regression models showed that protein had an inverse association with waist circumference. Saturated fatty acids and monounsaturated fatty acids were positively associated with triacylglycerol while polyunsaturated fatty acids had a negative association (Table 2).

**Table 2:** Multiple linear regression between MS components and energy, macronutrients and fiber of overweight and obese schoolchildren. Botucatu-SP-Brazil, 2007–2008

	MS components				
	WC	BP	HDL-C	TG	Glycemia
	β (SE)^a^				
Energy, macronutrients and fiber					
Total energy intake(kcal)	0.19 (0.21)	0.14 (0.25)	−0.25 (0.26)	0.45 (0.42)	0.03 (0.94)
Carbohydrates (g)	0.14 (0.13)	0.11 (0.18)	−0.14 (0.14)	0.34 (0.22)	0.03 (0.72)
Carbohydrates (%)	0.23 (0.79)	−0.19 (1.29)	0.76 (1.04)	0.24 (0.98)	−0.64 (5.55)
Protein (g/kg/day)	−*0.18 (0.04)* ^b^	−0.02 (0.08)	−0.04 (0.05)	0.08 (0.06)	0.02 (0.33)
Protein (%)	0.09 (0.07)	−0.06 (0.11)	0.04 (0.08)	−0.02 (0.08)	−0.17 (0.45)
Total fat (g)	0.06 (0.16)	0.29 (0.28)	−0.40 (0.24)	0.41 (0.25)	0.40 (1.12)
Total fat (%)	−0.84 (0.85)	0.60 (1.06)	−1.10 (1.02)	0.00 (0.76)	2.06 (4.49)
SFA (g)	0.02 (0.13)	0.19 (0.22)	−0.33 (0.17)	*0.47 (0.19)* ^c^	1.25 (0.99)
SFA (%)	−0.17 (0.13)	0.11 (0.20)	−0.18 (0.15)	0.22 (0.16)	1.44 (0.93)
MUFA (g)	0.04 (0.11)	−0.01 (0.18)	−0.14 (0.13)	*0.28 (0.14)* ^d^	−0.14 (0.85)
MUFA (%)	−0.07 (0.08)	−0.09 (0.12)	−0.01 (0.09)	0.03 (0.09)	−0.20 (0.53)
PUFA (g)	0.03 (0.11)	0.29 (0.18)	−0.07 (0.12)	−0.02 (0.13)	−1.10 (0.78)
PUFA (%)	−0.07 (0.09)	0.17 (0.15)	0.04 (0.10)	−*0.25 (0.12)* ^e^	−0.99 (0.65)
Cholesterol (mg)	−0.11 (0.36)	0.50 (0.62)	−0.44 (0.44)	1.05 (0.58)	4.81 (3.15)
Fiber (g)	0.37 (0.12)	0.09 (0.17)	−0.19 (0.12)	−0.22 (0.14)	−1.11 (0.75)

*SFA* saturated fatty acids, *MUFA* monounsaturated fatty acids, *PUFA* polyunsaturated fatty acids

^a^Regression model adjusted for age, sex, school, MS components. The values in italic corresponding of p value ≤ 0.05

^b^p value = 0.0003

^c^p value = 0.0144

^d^p value = 0.0467

^e^p value = 0.0317

**Table 3:** Multiple linear regression between MS components and food groups of overweight and obese schoolchildren. Botucatu-SP-Brazil, 2007–2008

	MS components				
	WC	BP	HDL-C	TG	Glycemia
	β (SE)^a^				
Food groups (servings)					
Cereals	0.42 (0.64)	−1.18 (1.07)	0.54 (0.74)	0.16 (0.78)	−*11.6 (5.50)* ^b^
Meats	0.91 (0.68)	−0.51 (0.96)	0.51 (0.71)	0.67 (0.77)	−2.38 (4.16)
Legumes	0.32 (0.17)	0.12 (0.26)	0.01 (0.18)	−*0.52 (0.23)* ^c^	−1.07 (0.92)
Vegetables	−0.10 (0.15)	0.32 (0.23)	*0.37 (0.18)* ^d^	0.23 (0.18)	0.00 (0.00)
Fruits	0.56 (0.49)	0.34 (0.73)	−0.25 (0.57)	0.69 (0.63)	0.00 (0.00)
Dairy products	−0.77 (0.64)	−0.22 (0.97)	−0.53 (0.73)	*2.84 (1.10)* ^e^	−4.11 (4.21)
Total energy intake (%)					
Sugars	−0.28 (0.19)	−0.03 (0.32)	0.06 (0.22)	0.29 (0.24)	0.42 (1.32)
Processed food	0.14 (0.19)	0.39 (0.33)	−0.11 (0.22)	0.22 (0.24)	*2.36 (1.16)* ^f^
Processed food + high sugar and fat content	0.27 (0.31)	−0.14 (0.54)	−0.41 (0.38)	*0.93 (0.42)*	*4.98 (2.54)*

^a^Regression model adjusted for age, sex, school, MS component. The values in italic corresponding of p value ≤ 0.05

^b^p value = 0.0356

^c^p value = 0.0247

^d^p value = 0.0451

^e^p value = 0.0103

^f^p value = 0.0451

^g^p value = 0.0288

^h^p value = 0.049

When using the MS components as outcome variables and food groups as predictor variables it was noted a positive association of vegetables consumption and HDL-C, and dairy food intake and triacylglycerol. A negative association was observed between cereal intake and glycemia, and legumes intake and triacylglycerol. Also, there was a positive association of processed foods intake and glycemia, and of processed food plus high sugar and fat content foods intake and glycemia and triacylglycerol (Table 3).

## Discussion

The main results of this study were: (a) the frequency of MS was 10.2 %, it was more frequent in obese schoolchildren and for schoolchildren with elevated body fat. The frequency were similar for both sex and for both sexual maturation stage (pre- and puberty). The MS percentage was low, but 62 % of schoolchildren showed at least one altered MS component; (b) the most frequent MS component was elevated waist circumference and the least one was glycemia. For all anthropometric measurements, except height, there was an increase according to the increase of number of MS components; (c) the MS was not associated with dietary intake, but its components were associated with some dietary factors. Triacylglycerol and glycemia showed more pronounced association with food components, mainly processed food and processed plus high sugar and fat content food. The saturated and monounsaturated fat were positively associated with triacylglycerol and the polyunsaturated fat was negatively associated with triacylglycerol. The legumes, especially beans, showed negative association with triacylglycerol.

In summary, our findings suggest that MS is not associated with dietary intake but when we analyzed MS components we found that plasma triacylglycerol and glucose concentrations had associations with food components such as high processed food and processed plus high sugar and fat content food.

The percentage of MS in this study was similar to regional Brazilian, American and European data from studies which adopted similar MS criteria [36–42], without influence of sex or sexual maturation. The most frequent MS components presented in our subjects were altered waist circumference followed by abnormal plasma concentrations of HDL-C and triacylglycerol. This same pattern was found in other studies [29, 41, 43–46]. A recent study with pre-pubertal Korean children found that altered waist circumference and abnormal blood pressure were the two most prevalent components of MS, followed by abnormal plasma concentration of triacyglycerol [19]. It is noteworthy that either altered waist circumference or BMI are the MS components with the highest prevalence in pediatric population. They also seem to be the differential factor of prevalence of MS among different studies, depending on the cutoff point adopted.

In our study, impaired glucose metabolism (fasting hyperglycemia) was detected in only one child, representing the MS component with the smallest frequency. This fact was also found in other studies [39, 40, 42, 44, 47, 48]. Such small percentage can be explained by the greater capacity of beta-pancreatic cells to compensate for excessive blood glucose in childhood. The investigation of plasma insulin concentration is indicated since it may be altered at this age range and represents a risk factor for MS [49]. Insulinemia has been assessed in epidemiological studies as an indicator of insulin resistance due to its high sensitivity and good correlation with HOMA-IR [50]. The altered glycated hemoglobin and hyperglycemia are more likely to be diagnosed only in children with BMI values above the 99th percentile, thus showing that glycemic alteration seems to be a late response to overweight [51]. It is discussed that the presence of [52] MS in children is more related to obesity, and in adolescence and adulthood to insulin resistance.

There are few studies performed with schoolchildren that associated dietary factors and MS. Liao et al. [53] found negative association between MS prevalence and healthy dietary score [53]. Some dietary characteristics such as not skipping main meals (breakfast, lunch and dinner), consuming less margarine and sweetened beverages were protective and decreased the development of MS [54] In the present study MS was not associated with dietary components and food group, probably because of the low MS percentage (10.2 %), also found by other studies [11–13, 55].

Triglyceridemia was positively associated with saturated fat and sugar intake, which are dietary items that are predominantly found in industrialized food, while polyunsaturated fat and legumes consumption were negatively associated [54, 56]. The main type of fatty acids found in processed food is palmitic acid or palmitate (C16:0). This fatty acid has been related not only to the larger production of adipose tissue (hyperplasia and hypertrophy), but also to larger production of oxygen reactive species and greater stimulus for TNFα production. Therefore, this inflammatory condition may lead to resistance to the peripheral action of insulin [57]. It is also postulated that high fat content, particularly that of saturated fat, leads to reduced peripheral and nervous-system leptin action and inhibition of insulin action in the brain, augmenting hunger and appetite [16].

In addition to saturated fat, industrialized food contain the *trans*-type fat, which may increase LDL-C concentration, reduce HDL-C, increase total cholesterol/HDL-C ratio, Lp(a) and reduce the size of the LDL-C particle, and make it more prone to oxidation [58]. A recent meta-analysis including only adults, showed that dietary cholesterol was not linearly related with plasma cholesterol and other lipids (LDL-C, HDL-C). The authors highlighted the positive association among saturate fats and the percentage of energy from total fat to cardiovascular diseases [59]. Trans fatty acids were not directly measured in the present study, but it was taken into consideration when assessing processed food intake and plasma lipid profile. An inverse relation between HDL-C and intake of food with processed plus high sugar and fat content food was noted in our study. Sanchez-Bayle et al. [60] also found a positive relation between total plasma cholesterol and saturated fat intake [60].

The intake of legumes in the present study is characterized by the intake of beans, which are considered to be a dietary source of fiber. Beans also contain soluble fibers in addition to 35 % of resistant starch [61, 62] and fructooligosaccharides, which are fermented in the small intestine, producing short-chain fatty acids that can reduce hepatic gluconeogenesis, thus resulting in better insulin resistance [16]. Soluble fibers delay gastric emptying and increase satiety and they can also reduce the absorption of biliary salts and increase their excretion, thus stimulating greater bile synthesis from endogenous cholesterol. These actions reduce the hepatic pool of cholesterol, and LDL-C receptors are increased as their clearance is increased. The actions of soluble fibers seem to be more evident for total cholesterol and LDL-C [61], but such mechanisms could maybe explain the inverse relation between legume intake and triacylglycerols found in this study. Ventura et al. [12] observed the relation between soluble fiber and a smaller number of components of the MS.

The present study found an inverse relation between plasma triacylglycerol and polyunsaturated fatty acid. Casazza et al. [63] also found an inverse relation between triacylglycerol and total fat; however, its type is not described. A possible explanation would be the presence of linolenic acid in polyunsaturated fat, which contributes to triacylglycerol reduction [64].

Several studies analyzing the association of BMI and waist circumference in children with energy intake have indicated that macronutrients of the diet may contribute to childhood obesity [65, 66]. In our study, waist circumference was inversely related to protein intake, which was also reported by Casazza et al. [63]. A higher protein intake was associated with greater weight loss in adolescents [67].

In the present study, it was found a positive association between glycemia and processed food with high sugar and fat content. A high intake of sugar and sugar-sweetened beverages is related to insulin resistance and reduced function of pancreatic beta-cells [68], and larger fat intake is related to insulin resistance [69]. A study with Brazilian adolescents showed a positive association between MS prevalence and ultra processed food [18]. The consumption of sugary beverages by adolescents showed a positive association with waist circumference and triacylglycerol [17]. The higher percentage of carbohydrate from sugar was associated with higher triacylglycerol, VLDL-C and HOMA-R. The increase of these lipids and insulin resistance indicators was higher for the sugar from sweetened beverages than from natural source [56].

The classification of industrialized food adopted in our study is similar to the criteria proposed by Monteiro et al. [70], that defines industrialized food as ready for consumption and different from the original raw-material. This is a very important classification of food, since the intake of such product has increased, particularly among children.

Our study had some limitations. The main limitation was related to the inclusion of the study sample criteria. We opted to include only overweight and obese schoolchildren because excess of weight is the main risk factor for MS, especially in the period when the study was being outlined (2007). We highlight the lack of a control group composed by eutrophic schoolchildren as an important limitation. If we had included a control group it would have been possible to investigate the different and the similar characteristics of dietary intake and whether the quality and amount of food would be associated with overweight or obese and MS components. Besides, it would be possible to explore if the MS components would be higher for overweight and obese children. However, a recent study showed low prevalence of MS in eutrophic schoolchildren using three widely used definitions specifically for children and adolescents. In this study obese schoolchildren showed the highest prevalence of MS [36]. Other three studies developed with schoolchildren in Europe and Asia showed that MS prevalence were higher in overweight and obese children [36, 71, 72].

Another limitation was that the highest percentage of refusal to participate in the study was from overweight children’s parents, especially from private school. The most usual explanation was that the parents did not agree with the diagnose of *overweight* and their children were already being accompanied by a pediatrician. The obese children’s parents agreed with diagnose of *obese* and they agreed to participate in the study. Some other limitation was to communicate subjects about the three 24-h dietary recalls in the beginning of the study. The parents and children could alter some dietary characteristics and they could bias the dietary intake and its relationship with MS components, but we had to be ethical and inform all the study phases before the parents agreed or not to participate. Before applying the 24-h recalls we asked if the child changed something in their dietary intake and all the answers were negative. Our work team also prepared some individual nutritional advice for parents who asked for it. We additionally had some lectures about healthy eating for parents in three schools (for all parents) and we referred all children with high blood pressure and altered biochemical exams for the pediatric clinic in Clinical Hospital of UNESP after the 4th phase of the study. Therefore, we believed that the previous explanation of three 24-h dietary recalls was not biased for the reasons above.

The last main limitation was dietary intake. It was necessary to use nutritional facts from industrialized food labels. In Brazil, for lipids, it is mandatory to show only total and saturated fat. Therefore, the intake of monounsaturated or polyunsaturated fat could be underestimated, particularly for the children who consumed large amounts of industrialized food. A Brazilian study showed nutritional information inadequacy on the labels of extruded snacks for saturated fat, fiber and sodium as well as cream filled sandwich cookies for saturated fats criteria [73]. These food types were largely consumed by the children in this study.

## Conclusions

The frequency of MS was low, but more than half of the subjects were diagnosed with at least one of its components. The obesity, diagnosed by BMI or body fat, was a positive indicator of MS. The waist circumference—the only anthropometric component—was the most frequent component of MS. The anthropometric measurements are relevant indicators for schoolchildren screening to biochemical examination and MS components. The triacylglycerol and glycemia were the most influenced MS components by dietary factors. Unprocessed food such as legumes, vegetables and cereals were considered protective dietary factors for lipid MS components and processed foods with high percentage of sugar and saturated fat were risk factor for lipids MS metabolic components.
